# Neurosteroids Alter p-ERK Levels and Tau Distribution, Restraining the Effects of High Extracellular Calcium

**DOI:** 10.3390/ijms252111637

**Published:** 2024-10-30

**Authors:** Vasiliki Konsta, Maria Paschou, Nikoleta Koti, Maria Evangelia Vlachou, Pantelis Livanos, Maria Xilouri, Panagiota Papazafiri

**Affiliations:** 1 Division of Animal and Human Physiology, Department of Biology, National and Kapodistrian University of Athens, University Campus, 15784 Athens, Greece; konstavasiliki@mail.ntua.gr (V.K.); mapasch@biol.uoa.gr (M.P.); nicoletakoti@biol.uoa.gr (N.K.); maria-evangelia.vlachou@univ-amu.fr (M.E.V.); 2 Division of Cell Biology, Department of Biology, Friedrich-Alexander University of Erlangen-Nuremberg, Staudtstrasse 5, 91058 Erlangen, Germany; panteleimon.livanos@fau.de; 3 Center of Clinical, Experimental Surgery & Translational Research, Biomedical Research Foundation of the Academy of Athens (BRFAA), 4, Soranou Efesiou Street, 11527 Athens, Greece; mxilouri@bioacademy.gr

**Keywords:** neurosteroids, allopregnanolone, dehydroepiandrosterone, Tau, ERK phosphorylation, Ca^2+^, mitochondria, actin cytoskeleton

## Abstract

Neurosteroids are undeniably regarded as neuroprotective mediators, regulating brain function by rapid non-genomic actions involving interference with microtubules. Conversely, hyperphosphorylated Tau is considered responsible for the onset of a plethora of neurodegenerative diseases, as it dissociates from microtubules, leading to their destabilization, thus impairing synaptic vesicle transport and neurotransmission. Consequently, we aimed to investigate the effects of neurosteroids, specifically allopregnanolone (Allo) and dehydroepiandrosterone (DHEA), on the levels of total and phosphorylated at Serine 404 Tau (p-Tau) in C57BL/6 mice brain slices. In total tissue extracts, we found that neurosteroids elevated both total and p-Tau levels without significantly altering the p-Tau/Tau ratio. In addition, the levels of several enzymes implicated in Tau phosphorylation did not display significant differences between conditions, suggesting that neurosteroids influence Tau distribution rather than its phosphorylation. Hence, we subsequently examined the mitochondria-enriched subcellular fraction where, again, both p-Tau and total Tau levels were increased in the presence of neurosteroids. These effects seem actin-dependent, as disrupting actin polymerization by cytochalasin B preserved Tau levels. Furthermore, co-incubation with high [Ca^2+^] and neurosteroids mitigated the effects of Ca^2+^ overload, pointing to cytoskeletal remodeling as a potential mechanism underlying neurosteroid-induced neuroprotection.

## 1. Introduction

The administration of neurosteroids is considered a promising therapeutic approach for various brain disorders [1]. Neurosteroids are neuroactive steroid hormones synthesized from steroidal precursors, the main being pregnenolone, in the mitochondria of neurons and glial cells, particularly in brain regions such as the cortex, hippocampus and amygdala [2,3]. They promote neuronal survival and modulate synaptic function primarily through allosteric modulation of neurotransmitter ionotropic receptors, such as γ-aminobutyric acid receptors type A (GABA_A_Rs), N-methyl-D-aspartate receptors (NMDARs) and α-amino-3-hydroxy-5-methyl-4-isoxazolepropionic acid receptors (AMPARs), thereby regulating neuronal excitability [4,5]. Allopregnanolone (Allo) and dehydroepiandrosterone (DHEA) are two important neurosteroids that bind to plasma membrane receptors (GABA_A_Rs and NMDARs, respectively) and modify inhibitory and excitatory neurotransmission, thereby regulating excitotoxic and apoptotic processes. In vivo studies have confirmed their protective role in models of traumatic brain injury and focal cerebral ischemia. In addition, in vitro studies have highlighted the effect of neurosteroids on cell signaling, with survival pathways being the most important [6]. Although scarce, there are indications that neurosteroids may also interact with the microtubule-associated protein 2 (MAP2) and other cytoskeletal elements [7].

The microtubule network is essential for proper neuronal function, mediating intraneuronal trafficking of synaptic vesicles and organelles and neuritic expansion. The microtubule-associated protein Tau (MAPT) contributes highly to the stabilization, organization and polymerization of microtubules. Recently, a new possible mode of Tau effect on the cytoskeleton has been proposed, suggesting that Tau accumulates in the labile domain of microtubules, displacing stabilizing proteins like microtubule-associated protein 6 (MAP6), thereby facilitating microtubule elongation [8]. In any case, depending on its subcellular location, Tau is also involved in multiple processes, including axial transport, cell polarity and neurotransmission [9]. Tau is a soluble, natively unfolded protein that maintains a highly flexible configuration and exhibits only a limited secondary structure [10]. Structural changes in Tau, such as hyperphosphorylation, often lead to its aggregation and accumulation in the brain, a hallmark of various clinically, biochemically and morphologically diverse neurodegenerative disorders known as “tauopathies” [11,12]. Hyperphosphorylation of Tau causes its detachment from microtubules and its subsequent intraneuronal aggregation into helical filaments and neurofibrillary tangles (NFTs). NFTs are key pathological features in Alzheimer’s disease and other tauopathies, though the mechanisms driving Tau hyperphosphorylation remain unclear [13]. Kinases and phosphatases that are linked with Tau pathogenesis are, inter alia, glycogen synthase kinase 3β (GSK-3b), cyclin-dependent kinase 5 (CDK5) and protein phosphatase 2A (PP2A) [14].

Modification of Tau-targeting protein kinases (GSK-3β, CDK5) revealed that Tau overexpression, hyperphosphorylation and aggregation significantly disrupt mitochondrial transport and distribution, often leading to synaptic dysfunction [15,16,17,18]. Moreover, Tau phosphorylated at Ser396-404 is enriched in the mitochondria of the hippocampal cells of elderly wild-type mice [19]. In fact, a stronger presence of this phosphorylated form is observed in synaptic mitochondria compared to non-synaptic, reinforcing hypotheses concerning the involvement of hyperphosphorylated Tau in age-related synaptic dysfunction and associated pathologies [20].

In addition, Ca^2+^ dyshomeostasis and related oxidative stress are common pathological triggers of Tau hyperphosphorylation [21]. Intraneuronal Ca^2+^ concentration [Ca^2+^] is tightly tuned, and its dysregulation can impair cellular functions and synaptic signaling, leading to neuronal loss and dementia [22]. Elevated [Ca^2+^] in mice brains has been shown to enhance Tau phosphorylation via a Ca^2+^-dependent increase in p25 [23]. Interestingly, hypercalcemia also disrupts intracellular Ca^2+^ homeostasis [24], with Ca^2+^ homeostasis modulator 1 (CALHM1), a non-selective voltage- and Ca^2+^-dependent cation channel, and calcium sensing by CaSR (calcium-sensing receptor) now recognized as key mediators of extracellular Ca^2^ effects on neuronal excitability and synaptic transmission [21,25].

Current understanding of the interplay between neurosteroids and Tau, two crucial components for neuronal survival and function, remains limited. The present study aimed to investigate the effects of Allo and DHEA on neuronal excitability, assessed by the levels of phosphorylated extracellular signal-regulated kinases (p-ERKs) as well as on Tau phosphorylation and distribution in brain slices from C57BL/6 mice. Our findings suggest that neurosteroids facilitate the localization of Tau protein forms near mitochondria and mitigate the effects of high extracellular [Ca^2+^], highlighting the pleiotropy of neurosteroids’ mechanisms of action.

## 2. Results

### 2.1. Neurosteroids Increase Basal p-ERK Levels Yet Attenuating Depolarization-Induced Phosphorylation of ERKs: An Effect More Pronounced in High [Ca^2+^]

**Figure 1:**
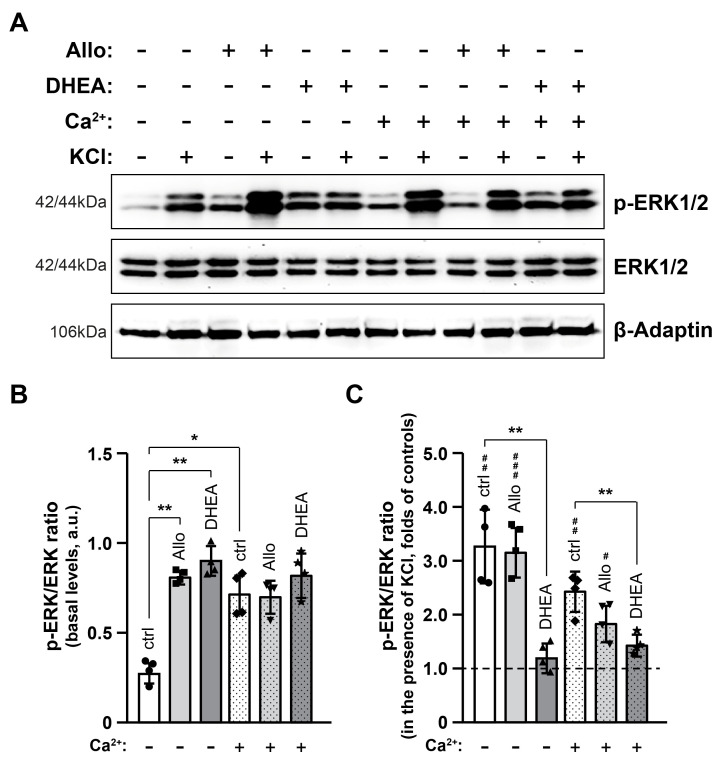
Mitigating effects of Allo and DHEA on depolarization-induced phosphorylation of ERKs. Total protein extracts (30 μg/lane) of brain slices were analyzed after 90 min incubation in the respective conditions by immunoblotting (**A**) for phosphorylated and total ERK levels, and the p-ERK/ERK ratio was quantified in un-depolarized (**B**) and KCl-treated samples (**C**). β-Adaptin was used as an internal loading control. The p-ERK/ERK ratio is expressed as the pixel intensity of p-ERK to the intensity of the corresponding total ERK and represents the phosphorylation state of ERKs. Data in (**B**) are presented in arbitrary units (a.u.) of the mean ± SD (n = 4), while in (**C**) relative to their respective undepolarized-control samples (set to 1, indicated with a dashed line). Geometric symbols represent individual measurements. Statistical significance was evaluated by one-way ANOVA followed by Tukey’s multiple comparison test (*, # *p* < 0.05; **, ## *p* < 0.01; ### *p* < 0.001). ERK1/2, extracellular signal-regulated kinase 1/2.

First, we tested brain tissue response to depolarizing conditions in the presence of Allo (10 μM), DHEA (10 μM) and/or elevated extracellular [Ca^2+^] (4 mM). To this end, cortical slices of adult male C57BL/6 mice were incubated for 90 min at 37 °C in an artificial cerebrospinal fluid (ACSF) solution containing the test compounds prior to a 5 min treatment with 45 mM KCl in ACSF (dACSF). Subsequently, phosphorylated and total levels of ERKs were analyzed by Western blot (Figure 1A). Our results revealed that basal p-ERK levels (Figure 1B) increased more than two-fold in the presence of either neurosteroids (*p* < 0.01) or high [Ca^2+^] (*p* < 0.05) compared to control conditions. However, these effects were not additive, as co-incubation of brain slices with high [Ca^2+^] and neurosteroids did not lead to a further increase in p-ERK levels.

To better illustrate the effects of experimental conditions on the depolarization potential, as assessed by p-ERK levels, we calculated the p-ERK/ERK ratio in the presence of KCl and divided it by the p-ERK/ERK ratio of the corresponding baseline level (in the absence of KCl). The p-ERK/ERK ratio of the basal levels was set to 1 (indicated by the dashed line in Figure 1C). KCl-induced membrane depolarization caused a more than 3-fold increase in ERK phosphorylation (*p* < 0.01) in control samples and those incubated with Allo (*p* < 0.001). On the contrary, slices incubated with DHEA did not exhibit any increase in p-ERK levels following depolarization. In samples treated with elevated [Ca^2+^], depolarization caused more than a two-fold increase in ERK phosphorylation (*p* < 0.01). Interestingly, in samples co-incubated with high [Ca^2+^] and Allo or DHEA, the depolarization-induced increase in ERK phosphorylation was lower than that caused by either factor alone, an effect which was more pronounced after incubation with DHEA (*p* < 0.01). These results indicate that neuronal excitability, as measured by ERK phosphorylation levels, is not affected by Allo, while DHEA alleviates KCl-induced p-ERK increase.

Next, we examined the levels of brain-derived neurotrophic factor (BDNF), given that this factor is known to modulate p-ERK levels during depolarization. Our results showed that the levels of both pro-BDNF and mature BDNF were similar across all conditions, and depolarization by KCl for 5 min did not result in any significant alterations in their levels, indicating that the observed immediate effects of KCl on neuronal excitability are independent of BDNF modulation (Figure S1). This highlights the complexity of the signaling mechanisms involved and the potential for neurosteroids to influence neuronal responses independently of BDNF dynamics during brief depolarization events.

**Figure 2:**
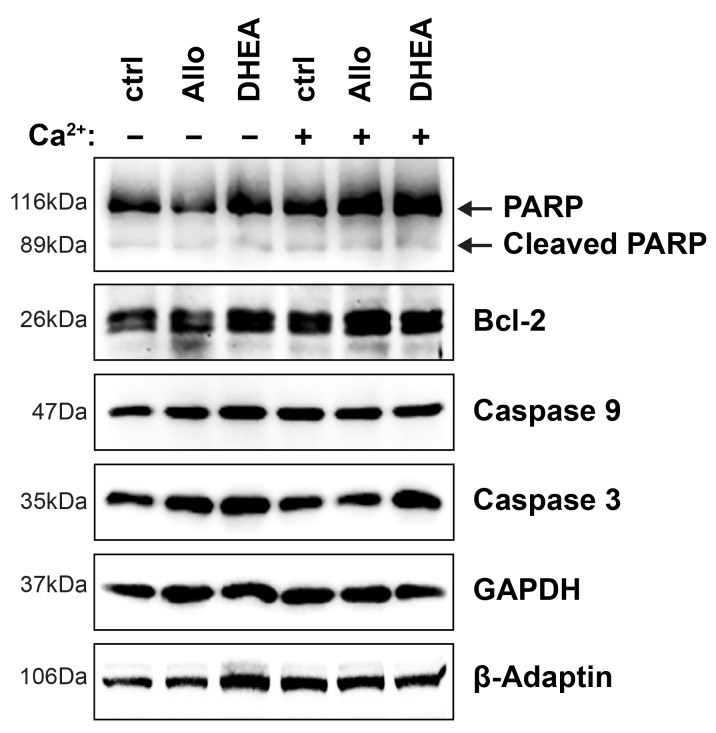
Neurosteroids and/or high [Ca^2+^] do not induce apoptosis. Total protein extracts (30 μg/lane) of brain slices were analyzed after 90 min incubation in the respective conditions by immunoblotting for PARP and its cleaved fragment, Bcl-2, caspases 3 and 9. Note that cleaved forms of caspases 3 and 9 were not detected. PARP, Poly (ADP-ribose) polymerase; Bcl-2, B-cell lymphoma 2.

Finally, to confirm that the aforementioned results were obtained from living and functioning tissue, we also assessed cell viability by measuring the levels of several proteins associated with cell death. Immunoblot analysis of Poly (ADP-ribose) polymerase (PARP), its cleaved fragment, B-cell lymphoma 2 (Bcl-2) and procaspases 3 and 9 and their cleaved fragments, showed no variability of their protein levels under the examined conditions (Figure 2). Collectively, these findings indicate that there is no tissue damage that could potentially confound our results, supporting the integrity of the experimental conditions.

### 2.2. Neurosteroids Increase Tau Levels but Attenuate Their Ca^2+^-Induced Rise

**Figure 3:**
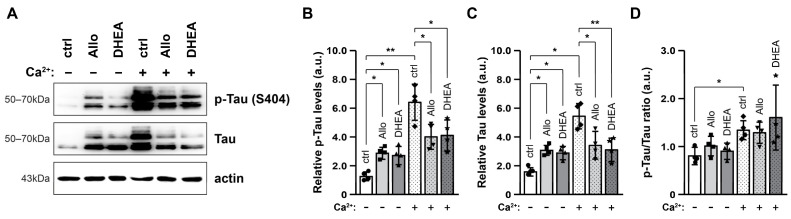
Neurosteroids and high [Ca^2+^] increase Tau protein levels. Representative immunoblots (**A**) and quantification of p-Tau (Ser404, **B**) and total Tau levels (**C**), and p-Tau/Tau ratio (**D**) in total protein extracts (30 μg/lane) after 90 min incubation of brain slices in the respective conditions. Actin was used as an internal loading control. All data are presented in arbitrary units (a.u.) of the mean ± SD (n = 4). Geometric symbols represent individual measurements. Statistical significance was evaluated by one-way ANOVA followed by Tukey’s multiple comparison test (* *p* < 0.05, ** *p* < 0.01).

We next examined the effects of Allo, DHEA and elevated extracellular [Ca^2+^] on total Tau and p-Tau (Ser404) levels in total protein extracts (Figure 3A). Our results showed that both p-Tau and total Tau levels were significantly increased in tissues incubated with either of the neurosteroids (Figure 3B,C, *p* < 0.05). However, analysis of the p-Tau/Tau ratio revealed that Tau phosphorylation levels remained stable (Figure 3D). On the other hand, elevated extracellular [Ca^2+^] exerted more pronounced effects, leading to further increases in p-Tau and Tau levels compared to control conditions (*p* < 0.01, *p* < 0.05, respectively). In this case, a small but statistically significant difference in the p-Tau/Tau ratio was observed (*p* < 0.05, Figure 3D), indicating an increase in Tau phosphorylation. Notably, neurosteroids significantly mitigated the Ca^2+^-induced increases in Tau and p-Tau levels, with DHEA demonstrating a more pronounced effect (*p* < 0.01) compared to Allo (*p* < 0.05). Nonetheless, the changes in the Tau phosphorylation state under these conditions remained non-significant. Collectively, among the conditions examined, a slight increase in Tau phosphorylation levels was detected only in the presence of high [Ca^2+^].

### 2.3. Tau-Modifying Proteins Are Not Significantly Affected by Neurosteroids and High Extracellular [Ca^2+^]

**Figure 4:**
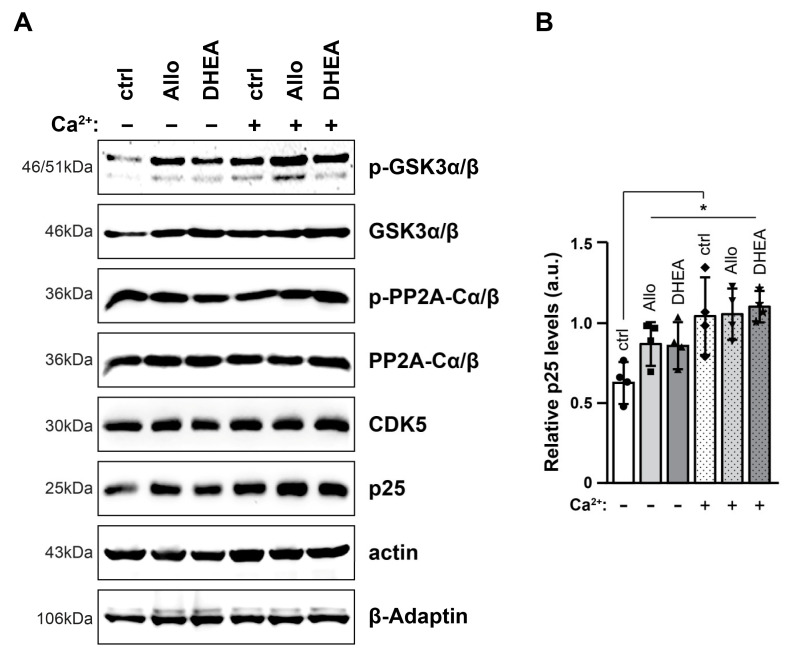
The levels of Tau-modifying proteins remain stable after incubation with neurosteroids and/or high [Ca^2+^]. Representative immunoblots of p-GSK3α/β, GSK3α/β, p-PP2A-Cα/β, PP2A-Cα/β, CDK5 and p25 in total protein extracts (30 μg/lane) (**A**). Relative p25 levels are expressed in arbitrary units (a.u.) of the mean ± SD (n = 4), based on the pixel intensity of p25 to the intensity of the loading control (**B**). Actin and β-Adaptin were used as internal loading controls. Geometric symbols represent individual measurements. Statistical significance was evaluated by one-way ANOVA followed by Tukey’s multiple comparison test (* *p* < 0.05). GSK-3b, glycogen synthase kinase 3β; PP2A-Cα/β, protein phosphatase 2A-Cα/β; CDK5, cyclin-dependent kinase 5.

Considering the multiple phosphorylation sites on Tau, we next sought to examine the levels of kinases and phosphatases known to interfere with Tau. Western blot analysis of total protein extracts revealed that the levels of most of the proteins examined, namely p-GSK3α/β, GSK3α/β, p-PP2A, PP2A and CDK5, remained unaffected by both neurosteroids and high extracellular [Ca^2+^] (Figure 4A). Only p25, a potent activator of CDK5, exhibited an increase across all conditions (*p* < 0.05) (Figure 4B).

We next sought to investigate whether the increase in Tau levels following incubation with neurosteroids is due to changes in gene expression. For this, we performed a quantitative RT-qPCR analysis using primers specific for murine (mmu) *MAPT* (Tau) mRNA, which showed that *Tau* mRNA levels remained stable in the presence of Allo and DHEA and/or high [Ca^2+^] (Figure S2). This result suggests that the elevated Tau levels may arise from other mechanisms, such as alterations in Tau protein stability, changes in its distribution or decreased degradation, rather than an increase in its synthesis.

### 2.4. Cytochalasin B Abolishes the Effects of Neurosteroids and High [Ca^2+^]

Based on the previous results, we assumed that Tau protein forms are enriched in the cytoplasm when exposed to neurosteroids and/or high extracellular [Ca^2+^]. We hypothesized that the interaction between Tau and actin cytoskeleton might be altered, prompting us to investigate Tau levels after co-incubating brain slices with cytochalasin B and neurosteroids or elevated [Ca^2+^]. Cytochalasin B inhibits actin polymerization by binding to the end of F-actin filaments. The chosen concentration of 1 mM was based on the literature data [26,27,28] and our preliminary experiments in which we tested the depolarization potential, assessed by p-ERK increases, at various concentrations of cytochalasin B. We found that the concentration of 1 mM is the maximum concentration that does not affect basal p-ERK levels while still enabling a significant increase in their levels during depolarization (Figure S3).

**Figure 5:**
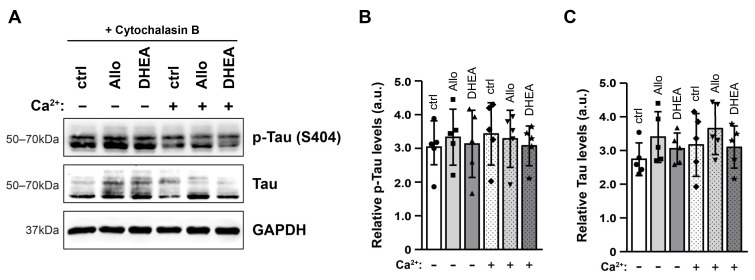
Cytochalasin B abolishes the effects of neurosteroids and high [Ca^2+^]. Representative immunoblots (**A**) and quantification of p-Tau (Ser404, **B**) and total Tau (**C**) levels in total protein extracts (30 μg/lane), using GAPDH as an internal loading control. All data are presented in arbitrary units (a.u.) of the mean ± SD (n = 5). Geometric symbols represent individual measurements. Statistical significance was evaluated by one-way ANOVA followed by Tukey’s multiple comparison test.

Although cytochalasin B did not affect the depolarization potential of brain slices in our experimental setup, it effectively abolished the effects of neurosteroids and high [Ca^2+^] on Tau levels. In particular, Western blot analysis of total protein extracts and subsequent quantitation revealed that the levels of Tau (total and p-Tau, Figure 5A) remained unchanged in the presence of cytochalasin B, indicating that binding of Tau to actin microfilaments is part of the cellular responses to neurosteroids or high extracellular [Ca^2+^].

### 2.5. Mitochondrial Marker VDAC Levels Are Not Affected by Neurosteroids and Elevated [Ca^2+^]

**Figure 6:**
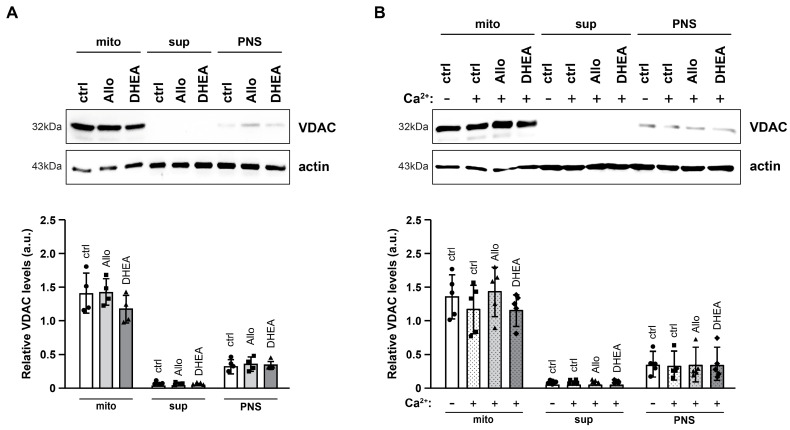
Subcellular fractionation and effects of neurosteroids on VDAC in the presence or absence of high extracellular [Ca^2+^]. (**A**,**B**) Representative immunoblots (**above**) and quantification (**below**) of VDAC on mitochondrial (mito), cytosolic (sup) and post-nuclear supernatant (PNS) fractions. Actin was used as an internal loading control. Relative VDAC levels are expressed in arbitrary units (a.u.) of the mean ± SD (n = 4). Geometric symbols represent individual measurements. Statistical significance was evaluated by one-way ANOVA followed by Tukey’s multiple comparison test. VDAC, Voltage-dependent anion channel.

Next, we performed subcellular fractionation to examine the potential differential translocation of Tau in the presence of neurosteroids and/or elevated extracellular [Ca^2+^]. We focused on the mitochondrial-enriched fraction due to Tau’s crucial role in the axonal transport of mitochondria, taking into consideration recent data showing the detection of Tau in mitochondria [19]. The fractionation process was validated by the differential presence of the mitochondrial membrane protein VDAC (Voltage-Dependent Anion Channel) (Figure 6). Immunoblot analysis of VDAC showed that this specific mitochondrial marker is faintly detected in the PNS fraction, which serves as the source for the subsequent mitochondrial-enriched and cytosolic fractions. Following analysis of the final fractions (mito, sup), VDAC was detected in significant levels only in the mitochondria-enriched fraction, confirming the effectiveness of the fractionation process.

Importantly, we did not observe any differences in VDAC levels across all fractions following incubation with neurosteroids and/or elevated [Ca^2+^]. Μitochondria are known to dynamically transport various pro- and anti-apoptotic proteins, and their selective release from the mitochondria is a crucial step in activating caspase 3. While we did not detect significant changes in apoptosis-related protein levels in total extracts (Figure 2), we further examined the levels of the pro-apoptotic protein Bcl-2-associated protein x (Bax) and the anti-apoptotic protein B-cell lymphoma-extra large (Bcl-xL) to determine if their relocalization had commenced in slices incubated for 90 min with neurosteroids and/or high [Ca^2+^]. Immunoblot analysis indicated that the levels of these proteins did not exhibit any significant changes under the examined conditions (Figure S4).

### 2.6. Neurosteroids Cause an Increase in Tau Levels in the Mitochondria-Enriched Fraction

**Figure 7:**
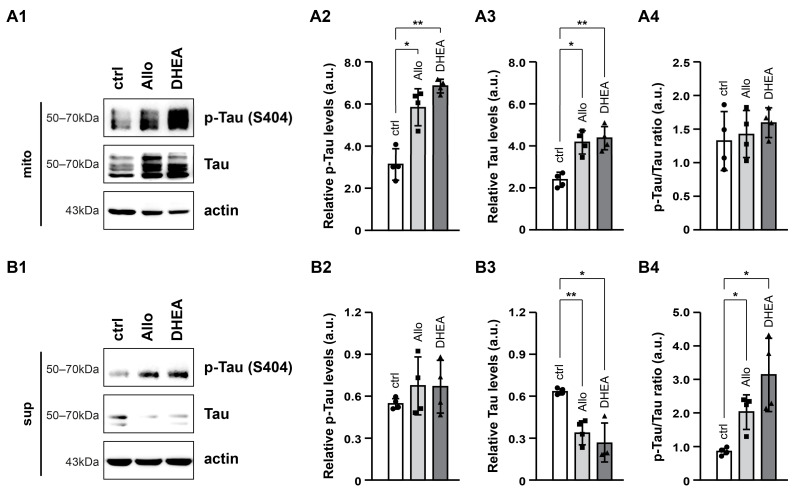
Effects of neurosteroids on p-Tau and Tau levels in mitochondrial and cytosolic fraction. Representative immunoblots (**A1**,**B1**) and quantification of p-Tau (Ser 404, **A2**,**B2**), Tau (**A3**,**B3**) and p-Tau/Tau ratio (**A4**,**B4**) in mitochondrial (mito, 10 μg/lane) and cytosolic (sup, 10 μg/lane) fraction. Actin was used as an internal loading control. The relative p-Tau and Tau levels are expressed as arbitrary units (a.u.) of the mean ± SD (n = 4) of the pixel intensity of p-Tau and Tau blot, respectively, to the corresponding intensity of actin. Geometric symbols represent individual measurements. Statistical significance was evaluated by one-way ANOVA followed by Tukey’s multiple comparison test (* *p* < 0.05, ** *p* < 0.01).

To assess the differential localization of Tau, we measured p-Tau and Tau levels, along with the p-Tau/Tau ratio in both the mitochondrial and the cytosolic fraction (Figure 7). In the mitochondrial fraction, both Allo (*p* < 0.05) and DHEA (*p* < 0.01) led to increased relative levels of p-Tau and Tau (Figure 7(A1–A3)), whereas Tau phosphorylation remained stable (Figure 7(A4)). Conversely, in the cytosolic fraction, Tau levels displayed a significant decrease in response to either of the neurosteroids (Figure 7(B1,B3)), while no significant differences were observed in p-Tau levels (Figure 7(B1,B2)). Consequently, the p-Tau/Tau ratio increased (Figure 7(B4)), likely due to the reduction in total Tau levels in the cytosolic fraction rather than an increase in Tau phosphorylation.

### 2.7. Neurosteroids Restrict the Ca^2+^-Induced Tau Increase

**Figure 8:**
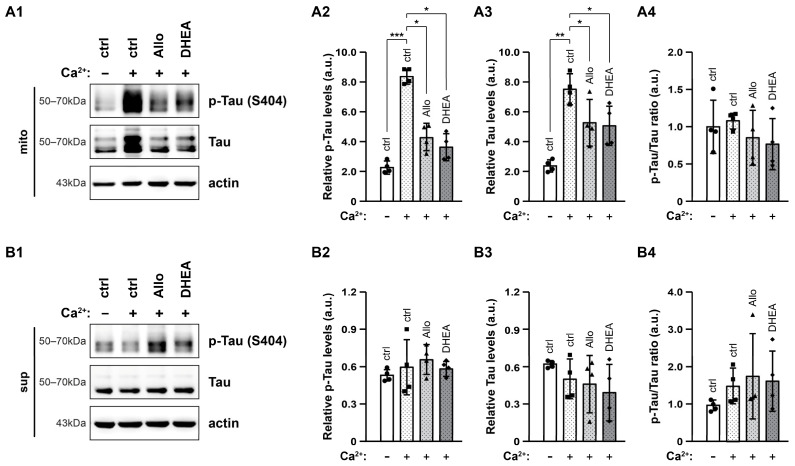
Effects of elevated [Ca^2+^] and neurosteroids on p-Tau and Tau levels in mitochondrial and cytosolic fraction. Representative immunoblots (**A1**,**B1**) and quantification of p-Tau (Ser 404, **A2**,**B2**), Tau (**A3**,**B3**) and p-Tau/Tau ratio (**A4**,**B4**) in mitochondrial fraction (mito, 10 μg/lane) and cytosolic fraction (sup, 10 μg/lane). Actin was used as an internal loading control. The relative p-Tau and Tau levels are expressed as arbitrary units (a.u.) of the mean ± SD (n = 4) of the pixel intensity of p-Tau and Tau blot, respectively, to the corresponding intensity of actin. Geometric symbols represent individual measurements. Statistical significance was evaluated by one-way ANOVA followed by Tukey’s multiple comparison test (* *p* < 0.05, ** *p* < 0.01, *** *p* < 0.001).

Lastly, we examined the effects of elevated [Ca^2+^] and neurosteroids on Tau levels across different subcellular fractions. In the mitochondria-enriched fraction, p-Tau levels were drastically augmented fourfold in the presence of high [Ca^2+^] (*p* < 0.001), an effect that was prevented by Allo and DHEA (Figure 8(A1,A2)). Tau displayed the same pattern, increasing in high [Ca^2+^] and decreasing in the presence of neurosteroids (*p* < 0.05) (Figure 8(A1,A3)), while its phosphorylation state remained stable (Figure 8(A4)). In contrast, in the cytosolic fraction, neither high [Ca^2+^] nor neurosteroids had an impact on p-Tau or Tau levels (Figure 8(B1–B4)).

## 3. Discussion

Neuronal function depends on the proper arrangement of microtubules, and their destabilization, for instance, due to Tau hyperphosphorylation, can lead to impaired intraneuronal trafficking and synaptic dysfunction [13,29,30]. Neurosteroids are known to protect neurons from excitotoxicity by modulating membrane neurotransmitter receptors, as well as excitatory signals [1,2,4,6]. The concentration of endogenous neurosteroids varies significantly, and their fluctuations are considered crucial for the regulation of many physiological functions in response to environmental and endogenous signals. While the concentrations of neurosteroids used during in vitro studies are rather high (1–20 μM) to facilitate direct binding to respective receptors, it is plausible that endogenous neurosteroid levels in the brain can also reach micromolar concentrations [31]. Interestingly, the brains of patients with Alzheimer’s disease exhibit abnormal steroidogenesis primarily attributed to mitochondrial dysfunction, as pregnenolone, the precursor of all neurosteroids, is synthesized there. In these patients, Allo levels were found to be significantly lower, whereas DHEA levels increased, possibly as a compensatory response to counteract Aβ-induced toxicity [32,33]. Therefore, neurosteroids are deemed as potential therapeutic agents, and their neuroprotective action in neurodegenerative diseases is of great interest. In this context, we assessed the effects of neurosteroids on the levels of total and phosphorylated, at Ser404, Τau in mouse brain slices utilizing elevated extracellular [Ca^2+^] as a potential neurotoxic insult.

First, we examined the levels of p-ERKs, which are established indicators of the depolarization potential of the plasma membrane and are activated by Ca^2+^ influx [34,35,36,37]. ERKs are members of the Mitogen-Activated Protein Kinase (MAPK) family, and their activation initiates a signaling cascade that leads to the activation of transcription factors, such as CREB (cAMP response-element binding protein), to the nucleus, thus regulating gene expression [38,39,40]. Once activated, ERKs can phosphorylate various substrates as well as nuclear and cytoskeletal elements, including Tau [39,40].

Our results suggest a distinct pattern of neurosteroid effects under resting versus depolarizing conditions. Specifically, we observed that basal levels of p-ERKs were increased in the presence of either Allo, DHEA or elevated extracellular [Ca^2+^]. On the contrary, upon depolarization, all treatments induced an increase in p-ERK levels, which was comparable to or smaller than control samples. Notably, DHEA alleviated the induced ERK phosphorylation. The effect of high extracellular [Ca^2+^] could be attributed to a continuous, subtle influx of Ca^2+^, as previously reported [24]. This ongoing Ca^2+^ influx could explain the increase in p-ERK basal levels while potentially shifting the membrane potential to a more positive value, thus resulting in ineffective KCl-induced depolarization. This reasoning might also apply to DHEA’s effects, given that DHEA acts as a positive regulator of NMDAR and enhances Ca^2+^ influx [4]. Regarding Allo, it is known to significantly increase Cl^−^ influx through GABA_A_ receptors [4,41,42], leading to membrane hyperpolarization. The observed increase in p-ERK basal levels in the presence of Allo seems paradoxical, as phosphorylated ERKs entail Ca^2+^ influx and depolarization of the plasma membrane. This discrepancy points to the existence of a mechanism of ERK phosphorylation that is independent of membrane depolarization. In fact, a crosstalk between GABA_A_Rs and GPCRs has been documented in neuronal cells [43], and since activation of GPCRs leads to ERK phosphorylation, it is plausible that p-ERK increase by Allo could be mediated by this crosstalk. Interestingly, this finding aligns with previous reports showing that Allo induces p-ERK levels in the ovariectomized rat hypothalamus [44]. ERKs are one of the downstream signaling substrates of BDNF, a neurotrophic factor that supports numerous aspects of neuronal survival and function, exhibiting neuroprotective effects [45]. BDNF levels are regulated by various activity-dependent elements, including CREB and, consequently, p-ERK itself, forming a positive feedback loop with a significant impact on neuronal biology. We did not observe significant effects of neurosteroids and high [Ca^2+^] on BDNF levels (Figure S1). More importantly, BDNF levels remained unchanged during the brief 5 min depolarization period, likely due to its short duration. Further investigation is needed to unravel the mechanisms behind these findings, as it is possible that a longer duration of depolarization might be required to observe significant changes. Such an approach, especially in the presence of different [Ca^2+^], would be of great physiological importance, given that hypocalcemia has been linked to hyperexcitability [46]. Overall, the finding that neurosteroids increase basal levels of p-ERKs while limiting their activation during depolarization may reflect their protective effects against hyperexcitability since controlled ERK activation promotes anti-apoptotic cellular pathways, preventing neurodegeneration [47]. There remains the possibility that these neurosteroids affect other signaling pathways, whether crosstalking with the ERK pathway or acting independently. For instance, both Allo and DHEA, administered at similar concentrations (for 24 h) to those used in this study, have been shown to attenuate inflammatory responses in mouse macrophage/monocyte RAW264.7 cells through the TLR (Toll-Like Receptor) pathway, also inhibiting the activation of LPS-stimulated AKT and NF-κB signaling [48,49].

Strikingly, a similar pattern was observed when analyzing Tau levels, revealing that both Tau and p-Tau (Ser404) levels were increased in the presence of neurosteroids or elevated [Ca^2+^]. However, these effects did not appear to be additive; rather, neurosteroids seemed to mitigate the effects of high [Ca^2+^]. Interestingly, the p-Tau/Tau ratio was significantly increased only in the presence of high extracellular [Ca^2+^], in accordance with previous studies indicating that part of Tau phosphorylation is likely mediated by Ca^2+^-dependent mechanisms [22,50,51,52]. Ca^2+^ influx activates calpain, which in turn is responsible for truncating p35 to p25, a more potent activator of CDK5 [53,54]. In the same direction, among the Tau-modulating proteins examined (GSK, PP2A, CDK5, p25), only p25 levels seemed to increase (Figure 4). Thus, despite the fact that CDK5 levels remained stable, its activity may be potentiated by the increased p25 levels, and it would be interesting to examine whether CDK5 mediates phosphorylation at sites other than S404.

Currently, little is known regarding the effects of neurosteroids on Tau phosphorylation. Only long-term treatments with progesterone and its metabolites have been shown to result in a significant decrease in the expression of Tau and GSK3β; however, in support of our findings, these treatments did not affect the levels of p-Tau on PHF-1 epitope (pSer396-pSer404) in the hypothalamus of rats [55,56]. Thus, all the above have led us to hypothesize that a different mechanism regulates Tau levels, namely, through the modulation of the cytoskeleton by neurosteroids, which may lead to the differential translocation of Tau.

Treatment of brain slices with cytochalasin B (Figure 5), a compound that depolymerizes actin [57], supports this notion. Our results showed that cytochalasin reverses any changes induced by neurosteroids or high [Ca^2+^], underscoring their modulatory effects on actin cytoskeleton. In support, neurosteroids can act as modulators of the microtubule cytoskeleton, capable of modifying neuronal cytoarchitecture, as both Allo and DHEA target two microtubule-associated proteins, MAP2 and cytoplasmic linker protein 170 (CLIP170) [4,5]. Additionally, they promote the disassembly of actin filaments, facilitating the detachment and translocation of vesicles. Moreover, both neurosteroids can activate intracellular signaling pathways that regulate cytoskeletal dynamics. For instance, NMDAR is found to directly interact with tubulin dimers and also influence microtubule dynamics by reducing MAP2 phosphorylation, thereby enhancing microtubular stability [58]. On the other hand, elevated intracellular [Ca^2+^] leads to microtubule destabilization and depolymerization by binding to Ca^2+^-binding sites in the microtubules GTP cap [59], thus intensifying Tau’s detachment and translocation into other subcellular compartments, including mitochondria.

Interestingly, Tau has been found to localize on both outer and inner mitochondria membranes [19,20]. Here, we demonstrated that in the presence of neurosteroids, Tau and p-Tau levels are increased in the mitochondria-enriched fraction, while both Allo and DHEA prevent Ca^2+^-induced increases in Tau levels. We speculate that neurosteroids exert this action, probably by interfering strongly with the cell’s cytoskeleton. It is possible that these neurosteroids can stabilize microtubules and inhibit their depolymerization, thus retaining Tau’s attachment to the microtubule network. This is of particular importance, given that Tau is known to inhibit the transport of organelles and promote retrograde translocation of mitochondria from neurites to the soma [60,61], which, in addition to its mislocalization from the axon to the soma and dendrites, lead to neuronal dysfunction and neurodegeneration. Furthermore, Tau hyperphosphorylation is associated with impaired trafficking and anchoring of NMDARs to the plasma membrane, ultimately resulting in synaptic dysfunction [62]. Therefore, it is proposed that this translocation may have neuroprotective effects, potentially ameliorating the toxic consequences of Tau accumulation.

The observation that Tau levels in the mitochondrial fraction are enhanced under the same conditions that also increase p-ERK levels may not be coincidental. It is possible that phosphorylation of ERKs initiates Tau’s translocation to the mitochondria. One possible mediator of this translocation could be p25, which was found to be elevated under conditions that also led to an increase in mitochondrial Tau, as p25 is upregulated through a MEK–ERK-dependent pathway [63]. Additionally, CDK5, activated by p25, is also a microtubular modulator, as it regulates tubulin acetylation, consequently enhancing microtubule stability [64]. Considering these interactions, it would be interesting to further investigate the emerging role of CDK5 on the microtubule cytoskeleton and its functional implications by assessing, for instance, the enzyme’s activity and levels in the mitochondrial fraction, elucidating its effects on microtubule dynamics and their relationship with Tau localization and function.

## 4. Materials and Methods

### 4.1. Mice Brain Slice Preparation

All experiments were conducted on C57BL/6 male mice 4–6 months old. Animals were housed in the animal facility of the Biomedical Research Foundation of the Academy of Athens (BRFAA) in a room with a controlled light-dark cycle and free access to food and water, as prescribed by the Greek Law 56/2013, in conformity with the European Union guidelines (2010/63). The animals used in the present study were not subjected to any experimental/special conditions before their sacrifice.

Brain slices from mouse brain hemispheres were used as a model system, as they maintain many features of their in vivo functions, and their preserved brain architecture allows interaction among diverse cell types and physiological synaptic connections. This model also enabled direct treatment with pharmacological compounds in a precisely controlled extracellular milieu. The dissection procedure was performed as described previously [65]. Briefly, mice were anesthetized using chloroform and then humanly decapitated. The brain hemispheres were placed in ice-cold cutting solution (2.14 mM KCl, 2.2 mM MgSO_4_, 27 mM NaHCO_3_, 1.47 mM NaH_2_PO_4_, 10 mM HEPES, 2 mM CaCl_2_, 10 mM Glucose, 200 mM Sucrose) for 10–15 min before being dissected into 400 μm thick slices using a microtome (McIlwain Tissue Chopper, Cavey Laboratory Engineering Co., Ltd., Gomshall Surrey, UK). Consequently, the slices were evenly distributed in six-well plates (3–4 slices per well) containing 5 mL of warm artificial cerebrospinal fluid (ACSF: 3.5 mM KCl, 1 mM MgSO_4_, 26 mM NaHCO_3_, 1.25 mM NaH_2_PO_4_, 126 mM NaCl, 10 mM HEPES, 2 mM CaCl_2_, 10 mM Glucose), the consistency of which resembles the natural cerebrospinal fluid, and were incubated for a 30 min equilibration period. After equilibration, the tissue slices were incubated in ACSF containing the examined compounds (10 μM Allopregnanolone, 10 μΜ Dehydroepiandrosterone and/or 4 mΜ [Ca^2+^]) for 90 min.

Following treatment, the ACSF was collected and centrifuged at 250× *g* for 5 min. The pellet, containing cells that had leaked from the slices, was resuspended and incubated in PBS containing 0.1% trypan blue for at least 3 min. The death rate was determined as the percentage of dead cells relative to the total number of cells. A total of 150–200 cells were counted across three to four subfields of each sample. Cell counts were performed by two investigators who were unaware of the respective treatments to eliminate bias and ensure that the results were objective.

Slices that were intended to undergo depolarization were incubated with depolarization buffer (dACSF: 45 mM KCl, 1 mM MgSO_4_, 26 mM NaHCO_3_, 1.25 mM NaH_2_PO_4_, 84.5 mM NaCl, 10 mM HEPES, 2 mM CaCl_2_, 10 mM Glucose) for 5 min before the end of the incubation period, and the reaction was terminated immediately by washing the slices with ice-cold ACSF.

### 4.2. Tissue Homogenization

Whole tissue homogenization was performed as described previously [66]. Briefly, tissue samples were mechanically homogenized in 300 μL TNE Lysis Buffer [50 mM Tris-HCl, pH 7.6, 2 mM EDTA, 150 mM NaCl, 1% SDS, protease inhibitors and phosphatase inhibitors (20 mM NaF, 20 mM β-glycerophosphate)] using 22-gauge syringes (5–10 passes) and incubated for 30 min at 100 °C. The homogenates were subsequently centrifuged at 13,000 rpm for 10 min at room temperature. The supernatants were collected into new tubes and stored at −80 °C until use.

### 4.3. Subcellular Fractionation

The slices were transferred in 500 μL ice-cold mitochondria isolation buffer (MIB: 10 mM HEPES pH 7.5, 0.25 mM Sucrose) and incubated on ice for 10 min. Tissue samples were then homogenized using a Dounce homogenizer (30 strokes) and centrifuged at 6000 rpm for 10 min at 4 °C. This step yielded the post-nuclear supernatant (PNS) in the supernatant and whole cells, membranes and nuclei in the pellet. A volume of 50 μL of the PNS was transferred to new tubes, while the remaining supernatant was centrifuged once more at 12,000 rpm for 6 min at 4 °C. The resulting pellet represented the mitochondrial fraction, and the supernatant constituted the cytosolic fraction, to which 1% Triton X-100 was added before transferring into new tubes. The mitochondrial fraction was washed with MIB, centrifuged and then resuspended in 100 μL mitochondria resuspension buffer (300 mM NaCl, 10 mM CaCl_2_, 100 mM Tris-HCl pH 8.5, 0.5% NP40) and incubated for 30 min at 4 °C.

### 4.4. Western Blot

A Pierce BCA Protein Assay Kit (Thermo Fisher Scientific, Waltham, MA, USA) was used for the quantification of total protein amounts.

Equal amounts of protein, from total extracts as well as mitochondrial and cytosolic protein extracts, were separated by SDS-PAGE electrophoresis on 12–14% polyacrylamide gels under denaturing conditions and then transferred to nitrocellulose membranes (Porablot NCP; Macherey-Nagel, Düren, Germany) using a wet tank transfer system (BioRad Laboratories, Richmond, CA, USA). The membranes were subsequently blocked for 1 h at room temperature with Tris-buffered saline (TBS) containing 5% powdered milk and 0.1% Tween-20. Afterwards, the membranes were probed with the respective primary (overnight at 4 °C) and secondary (2 h incubation at room temperature) antibodies. All antibodies were diluted in TBS containing 5% BSA and 0.1% Tween-20 (a list of the antibodies used in this study and their dilution ratios can be found in Table S1). The visualization of the immunoreactive bands was based on the Enhanced Chemiluminescence (ECL) method using the Luminata™ Crescendo Western HRP Substrate (Millipore, Billerica, MA, USA) and the 8800 FluorChem Imaging System (Alpha Innotech Corp., San Leandro, CA, USA). All uncropped western blot images are provided in Figure S5.

### 4.5. Total RNA Isolation and RT-qPCR Analysis

Total RNA from brain slices was extracted using the TRIzol^®^ Reagent according to the manufacturer’s protocol (Thermo Fisher Scientific). Briefly, the first strand of cDNA was synthesized from 1 μg total RNA using random hexamer primers, according to the M-MLV reverse transcriptase protocol (Thermo Fisher Scientific). qPCR assay was based on SYBR^®^ Green I DNA binding dye (Thermo Fisher Scientific) and was carried out in 96-well PCR microplates (Applied Biosystems, Forster City, CA, USA) on a 7500 Real-Time PCR System (Applied Biosystems). Fluorescence emission of the PCR products and subsequent quantification were carried out with the 7500 Real-Time PCR Software v2.3 (Applied Biosystems). The reaction mixture (20 μL total volume per well) included 9 μL of 10× diluted cDNA (~20 ng), 9.6 μL Kapa SYBR^®^ Fast Universal 2x qPCR Master Mix (Kapa Biosystems, Roche, Basel, Switzerland), 0.4 μL of 50x Rox Low passive reference dye (Kapa Biosystems) and primers at a final concentration of 200 nmol/L. Each qPCR run included a no-cDNA template control and reverse transcription negative controls. All negative controls yielded no or minimal detectable quantification cycle value, proving the lack of any contamination or non-specific signal. Each sample was tested in triplicates, and data obtained from three independent experiments were analyzed using the 2^−ΔΔCT^ method, using *GAPDH* and *U6* mRNAs as normalization standards. Primer sequences for RT-qPCR are shown in Figure S2A.

### 4.6. Statistical Analysis

The protein levels were normalized, using the housekeeping proteins actin, β-adaptin or GAPDH as loading controls. The intensity of each immunoreactive band was estimated by densitometric quantification using the Fiji ImageJ software [version 2.9.0; accessed on 1 April 2024; https://fiji.sc/, National Institute of Health (NIH), Madison, WI, USA]. Specifically, the levels of the protein of interest were quantified and normalized to their respective loading controls (actin, GAPDH, β-Adaptin) by dividing the pixel intensity of the examined protein by the intensity of the corresponding loading control. For the phosphorylation status (ratio) of proteins (e.g., Tau, ERK1/2, GSK3α/β, PP2A-Cα/β), we quantified the pixel intensity of both the phosphorylated and total forms of the protein. Each form was then normalized to its respective loading control, and the ratio of the phosphorylated/total form was calculated by dividing the normalized phosphorylated protein level by the normalized total protein level. The resulting values were expressed as arbitrary units (a.u.) derived from these calculations for both control and treated samples. In some cases, the control values were set to 1, and data were expressed relative to the control.

All data are presented as the mean ± standard deviation (SD) of at least three independent experiments. Depending on the different groups examined, comparisons were carried out using either the Student’s *t*-test or one-way ANOVA followed by Tukey’s multiple comparison test. Statistical analysis was performed using GraphPad Prism Software (version 9.0.0; San Diego, CA, USA), and the threshold for statistically significant differences was set to probability values (*p*) less than 0.05.

## 5. Conclusions

In the present study, we report that short-term treatment of mouse brain slices with elevated concentrations of the neurosteroids Allo and DHEA increases the basal levels of p-ERKs and of extractable Tau protein, with the latter being particularly abundant in the mitochondria-enriched fraction. Furthermore, Tau’s translocation and enrichment in the mitochondrial fraction in the presence of neuroprotective agents may indicate a novel, non-neurotoxic role for this protein. Interestingly, these neurosteroids attenuate the pronounced effects of high extracellular [Ca^2+^], signifying their protective role in neuronal cells. Although the exact mechanism remains to be elucidated, our findings point to an actin-dependent mechanism on Tau redistribution. Importantly, it seems that the effects of neurosteroids on actin cytoskeleton drive to a conformation that counteracts the effects of elevated [Ca^2+^]. These findings affirm the neuroprotective properties of neurosteroids, deeming them as potential therapeutic agents against neurodegeneration.

## Data Availability

The data presented in this study are available on request from the corresponding author.

## Supplementary Materials

The following supporting information can be downloaded at: https://www.mdpi.com/article/10.3390/ijms252111637/s1.

## Abbreviations

ACSF, Artificial cerebrospinal fluid; Allo, Allopregnanolone; Bax, Bcl-2-associated protein x; Bcl-2, B-cell lymphoma 2; Bcl-xL, B-cell lymphoma-extra large; BDNF, Brain-derived neurotrophic factor; CDK5, Cyclin-dependent kinase 5; DHEA, Dehydroepiandrosterone; ERK 1/2, Extracellular signal-regulated kinase 1/2; GABA_A_R, γ-Aminobutyric acid receptor type A; GPCR, G-protein coupled receptors; GSK-3α/β, Glycogen synthase kinase 3α/β; MAP2, Microtubule-associated protein 2; MAPK, Mitogen activated protein kinase; MAPT, Microtubule-associated protein Tau; NFT, Neurofibrillary tangle; NMDAR, N-methyl-D-aspartate receptor; PARP, Poly (ADP-ribose) polymerase; PP2A, Protein Phosphatase 2A; VDAC, Voltage-dependent anion channel.
