# Supplementary material for: Neurosteroids Alter p-ERK Levels and Tau Distribution, Restraining the Effects of High Extracellular Calcium
Source: Int J Mol Sci. 2024 Oct 30;25(21):11637. doi: 10.3390/ijms252111637 (PMC11546054; doi:10.3390/ijms252111637)
Supplement: Supplementary file 1 [file ijms-25-11637-s001.zip › Table S1.pdf]

Table S1. Primary and secondary antibodies used in Western blot.

Primary Antibodies	Manufacturer	Catalog No.	Purification	Host	Dilution	MW (kDa)
β-Adaptin	Santa Cruz Biotechnology (Santa Cruz, CA, USA)	sc-6425	mAb	Goat	1:2000	106
GAPDH		sc-365062	mAb	Mouse	1:2000	37
phospho-PP2A-Cα/β (Tyr307)		sc-12615-R	pAb	Rabbit	1:200	36
PP2A-Cα/β		sc-6110	pAb	Goat	1:500	36
mature BDNF/pro-BDNF		sc-33904	pAb	Goat	1:750	14, 32
Actin	Millipore (Billerica, MA, USA)	MAB1501	mAb	Mouse	1:1000	43
phospho-GSK3α/β (Ser219)	Cell Signaling Technology (Boston, MA, USA)	#9331	pAb	Rabbit	1:1000	46, 51
GSK3β		#9315	mAb	Rabbit	1:1000	46
phospho-p44/42 MAPK [p-ERK1/2 (Thr202/Tyr204)]		#9101	pAb	Rabbit	1:1000	42, 44
p44/42 MAPK (ERK1/2)		#9102	pAb	Rabbit	1:1000	42, 44
Tau		#46687	mAb	Rabbit	1:1000	50-70
VDAC		#4661	mAb	Rabbit	1:1000	32
PARP (full length & cleaved)		#9542	pAb	Rabbit	1:1000	116, 89
Caspase 3 (procaspase & cleaved)		#9662	pAb	Rabbit	1:500	17, 19, 35
Caspase 9 (procaspase & cleaved)		#9508	mAb	Mouse	1:1000	35, 37, 47
Bax		#2772	pAb	Rabbit	1:750	20
Bcl-XL		#2764	mAb	Rabbit	1:750	30
Cytochrome c		#4272	pAb	Rabbit	1:750	14
Bcl-2	BD Transduction Laboratories	#846520	mAb	Mouse	1:750	26
phospho-Tau (Ser404)	OriGene (Rockville, MD, USA)	AP02404PU-N	pAb	Rabbit	1:1000	50-70
CDK5		TA500397	mAb	Mouse	1:1000	30
p25	Kind gift from Prof. Jensen PH, University of Aarhus, Denmark	-	mAb	Mouse	1:1000	25
**Secondary Antibodies**						
HRP-conjugated anti-rabbit IgG	R&D Systems (Oakville, Canada)	#HAF008	pAb	Goat	1:1000	
HRP-conjugated anti-mouse IgG	Millipore (Billerica, MA, USA)	#AP124P	pAb	Goat	1:1000	
HRP-conjugated anti-goat IgG		#AP106P	pAb	Rabbit	1:1000	
