# Supplementary figures and images for: Neurosteroids Alter p-ERK Levels and Tau Distribution, Restraining the Effects of High Extracellular Calcium
Source: Int J Mol Sci. 2024 Oct 30;25(21):11637. doi: 10.3390/ijms252111637 (PMC11546054; doi:10.3390/ijms252111637)

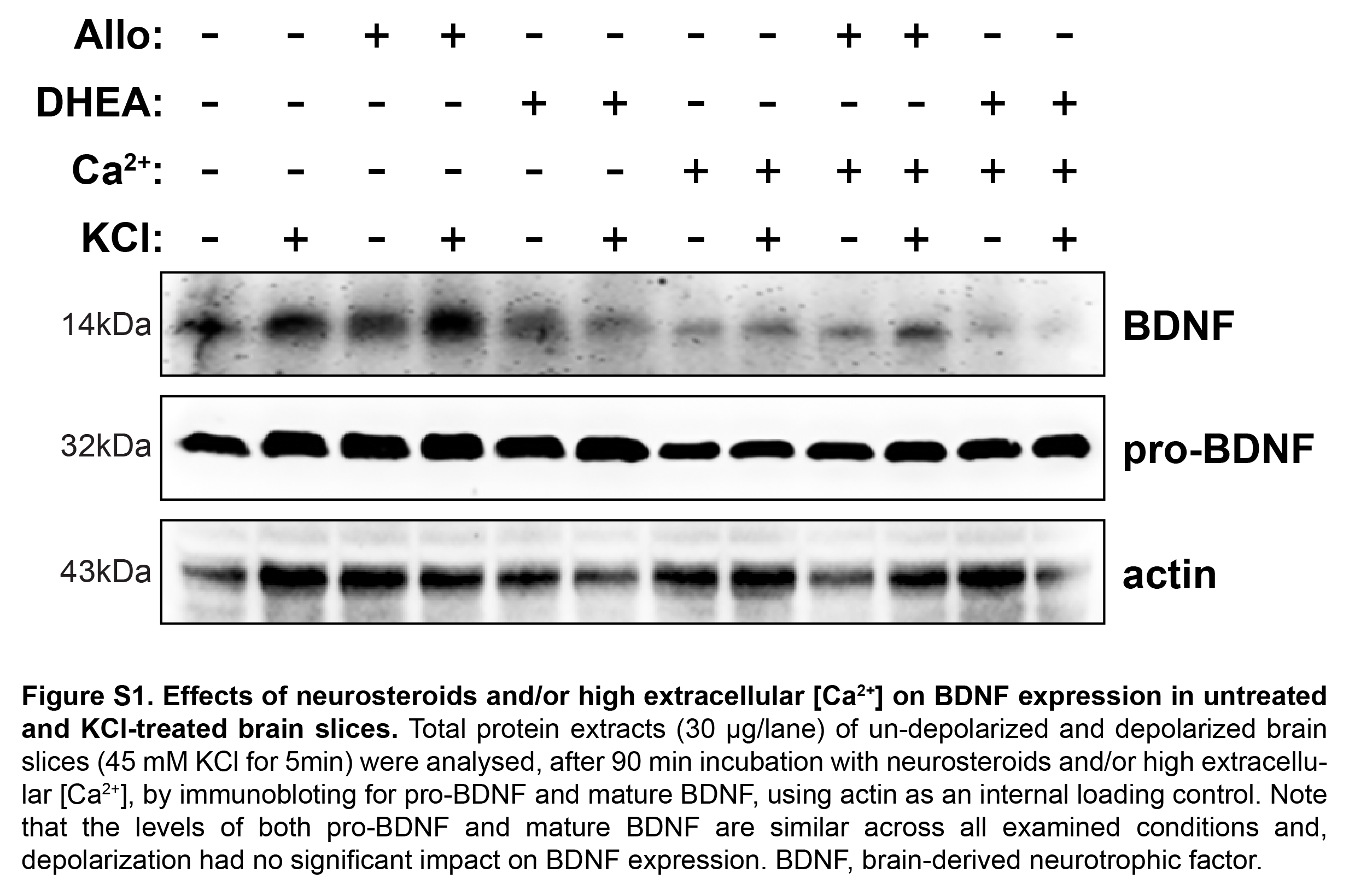

Supplement: Supplementary file 1 [file ijms-25-11637-s001.zip › Figure S1.tif]

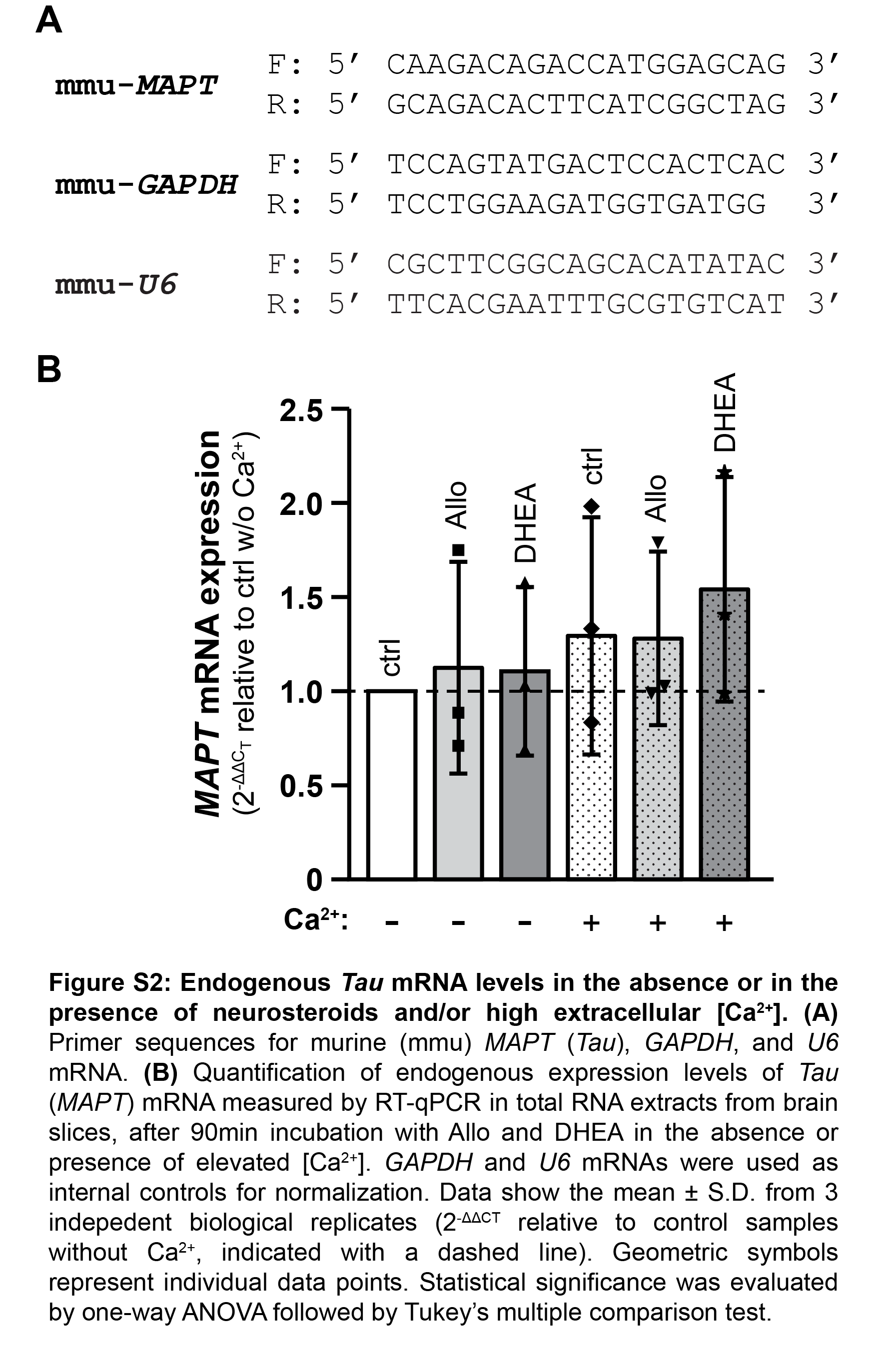

Supplement: Supplementary file 1 [file ijms-25-11637-s001.zip › Figure S2.tif]

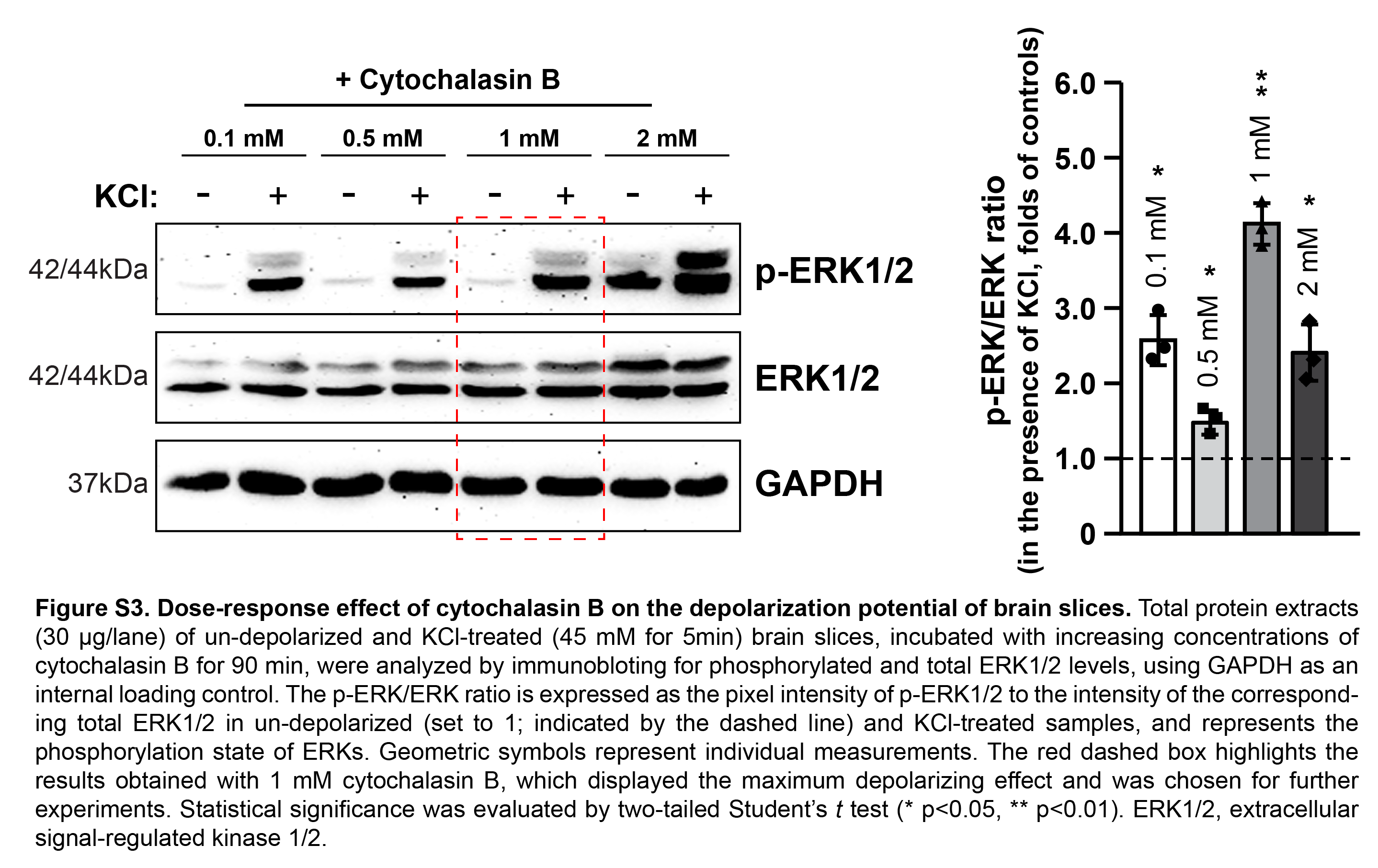

Supplement: Supplementary file 1 [file ijms-25-11637-s001.zip › Figure S3.tif]

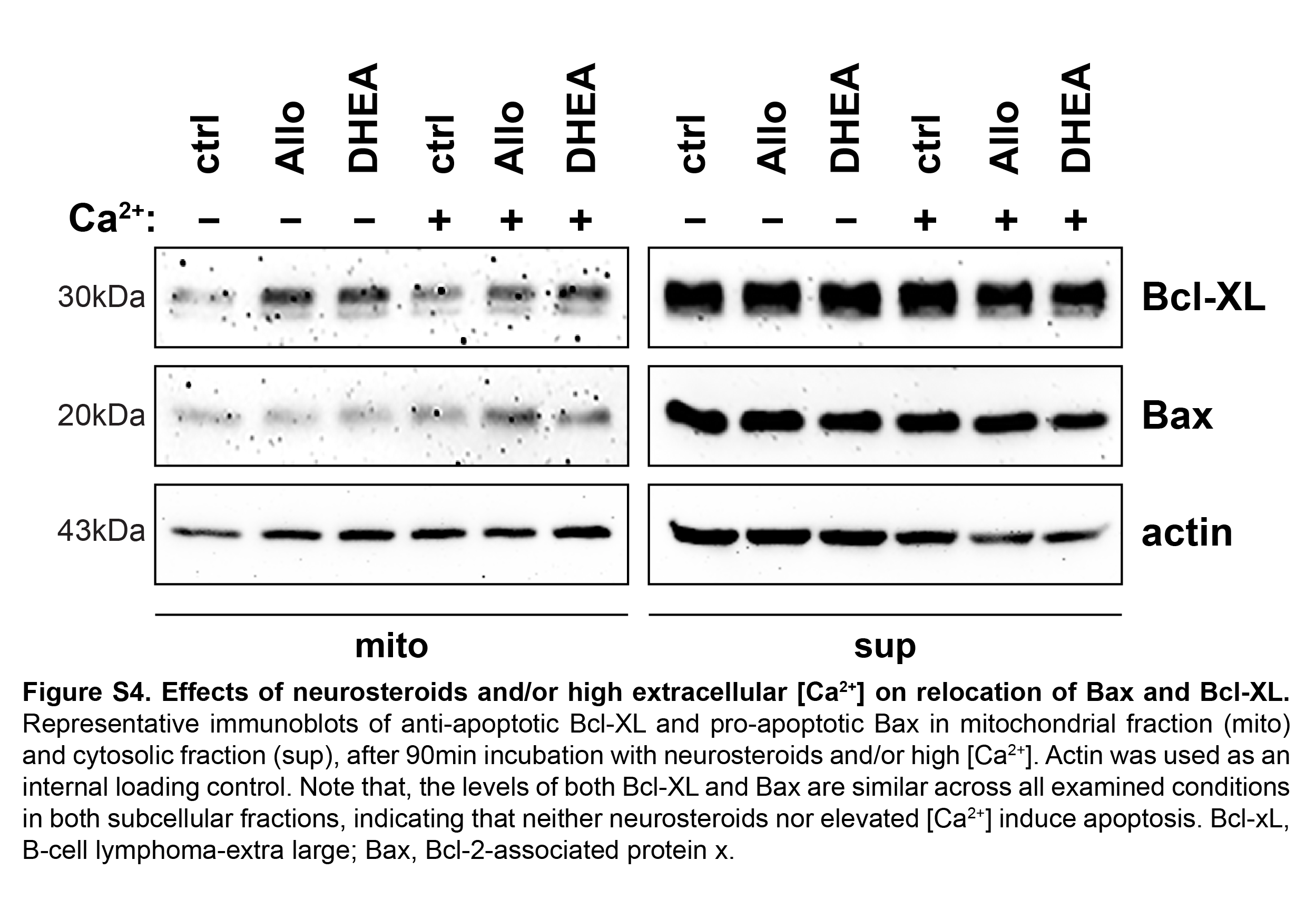

Supplement: Supplementary file 1 [file ijms-25-11637-s001.zip › Figure S4.tif]
